# Comprehensive evaluation of morphological and physiological responses of seventeen *Crassulaceae* species to waterlogging and drainage under temperate monsoon climate

**DOI:** 10.1186/s12870-023-04676-z

**Published:** 2024-01-02

**Authors:** Jie Zhang, Feng Song, Xiaolei Xu, Tiantian Xia, Xu Zhang, Li Dong, Dejie Yin

**Affiliations:** 1 Shandong Jianzhu University, Jinan, China; 2 Beijing Key Laboratory of Ornamental Plants Germplasm Innovation & Molecular Breeding, National Engineering Research Center for Floriculture, Beijing Laboratory of Urban and Rural Ecological Environment, School of Landscape Architecture, Beijing Forestry University, Beijing, 100083 China

**Keywords:** Waterlogging, *Crassulaceae*, Adventitious roots, Antioxidant enzymes

## Abstract

Unpredictable rainfall frequently results in excess moisture, which is detrimental to the landscape because it interferes with the genetic, morphological, and physiological processes of plants, even though the majority of urban landscapes frequently experience moisture shortages. A study was conducted to analyze the effects of a 36-day waterlogging phase and a subsequent 12-day recovery period on the morpho-physiological responses of 17 *Crassulaceae* species with the goal of identifying those which were more tolerant of the conditions. Results revealed that waterlogging stress has an impact on all morpho-physiological parameters. Sensitive materials (S7, *Hylotelephium telephium* ‘Purple Emperor’ and S15, *S. sexangulare*) showed severe ornamental quality damage, mortality, decreases in total dry biomass, root-shoot ratio, and chlorophyll content, as well as higher MDA concentrations. Lower reductions in these parameters, along with improved antioxidant enzyme activities and greater recovery capabilities after drainage, were observed in the most tolerant materials S2 (*H. spectabile* ‘Brilliant’), S3 (*H. spectabile* ‘Carl’), and S5 (*H. telephium* ‘Autumn Joy’). Furthermore, with the exception of early death materials (S7 and S15), all materials showed varying intensities of adventitious root formation in response to waterlogging. The 17 species were divided into 4 clusters based on the comprehensive evaluation value. The first group included S1-S3, S5-S6, S8-S12, which were waterlogged tolerant with the highest values (0.63–0.82). S14 belongs to the intermediate waterlogging tolerant. S4, S13, S16, and S17 were clustered into the low waterlogging-tolerant group. S7 and S15 were the most susceptible to waterlogging. The survival and success of *Crassulaceae* species (especially, the first and second cluster), throughout this prolonged period of waterlogging (36 days) and recovery were attributed to a combination of physiological and morphological responses, indicating that they are an appealing species for the creation of rain gardens or obstructed drainage locations.

## Introduction

Waterlogging has been identified as one of the most significant abiotic stresses that negatively impact the development, distribution, productivity, and survival of vegetation worldwide, including agricultural and horticultural systems [1, 2], as well as natural ecosystems [3, 4]. According to the AR6 synthesis report on Climate Change 2023, waterlogging is expected to become more common and severe in certain regions in the coming decades [5]. Moreover, a temperate monsoon climate region with hot, wet summers and chilly, dry winters is more prone to waterlogging. Understanding how vegetation affected by waterlogging responds both structurally and functionally is crucial.

Under waterlogging circumstances, oxygen shortage in the soil environment is caused by rapid consumption of O_2_ and decreased rates of gas exchange with the atmosphere at the soil surface [1]. Anaerobic fermentation replaces aerobic respiration as a result of severe soil hypoxia or even anoxia, which is directly affects by the root system [6, 7]. Numerous plant processes, including gene expression [8], energy consumption [2], cellular metabolism [9, 10], carbohydrate reserves, and translocation [11] are negatively impacted by the accumulation of phytotoxic glycolysis byproducts and the decrease in pH and redox potential. These factors have a significant impact on the ability of plants to survive under these circumstances [12–14].

Plants that experience waterlogging frequently show signs of leaf withering, leaf chlorosis, and leaf abscission [14, 15]. Plants in waterlogged conditions clearly exhibit significant reductions in leaf area, biomass allocation, photosynthetic rate, and even eventual death, especially in species that are sensitive to water [16–19]. The decline or low concentrations of Chl have been described as a long-term reaction and one of the unique characteristics of waterlogging, causing a loss in leaf photosynthetic capacity [3, 11]. Stomata closure, decline in leaf chlorophyll content, early leaf senescence, and reduced leaf area all contribute to the reduction of photosynthesis during root hypoxia [14, 20]. Damage to mesophyll cells is also responsible for later restrictions in photosynthesis [15, 21]. To forecast, monitor, and detect stress in plants, chlorophyll fluorescence is frequently employed as a indicator of the photochemical efficiency of Photosystem II (PSII). It can also be used as a more general marker of how plants respond to environmental change [22]. Plants may exhibit a variety of morphological, physiological, and metabolic adaptations in response to soil flooding, despite the presence of stress symptoms [3, 14].

Plants’ key morphological and anatomical responses to root hypoxia include not only hypertrophied lenticels and adventitious roots, but also the development of aerenchyma and a radial oxygen-loss barrier [2, 6, 23, 24]. All of these adaptations induced by waterlogging stress aid in the capture and diffusion of oxygen [24], the release of phytotoxic compounds produced during anaerobic metabolism [25], and the maintenance of water and nutrient uptake [26], thereby minimizing the effects of flooding on shoot physiological activity.

Plants have mechanisms to cope with this stress, in addition to the adaptations mentioned above. These mechanisms include increased availability of soluble sugars, increased activity of glycolytic pathway and fermentation enzymes, and involvement of antioxidant defense mechanisms to cope with post-hypoxia/anoxia oxidative stress [27, 28]. Soluble sugar acts as an osmotic agent or osmoprotector, protecting the structural integrity of proteins and membranes under abiotic circumstances [11, 27, 29, 30]. In fact, the quantity of stored carbohydrates and their accessibility may distinguish between species that are tolerant and intolerant. Long-term low-oxygen stress can disrupt cellular homeostasis by inducing peroxidative reactions and causing oxidative damage to various cellular components, such as phospholipids, proteins, and nucleic acids. These effects can include a loss of enzyme activity, altered membrane fluidity, genomic damage, and even cell death [31]. An endogenous antioxidant defense system, composed of antioxidative enzymes such as superoxide dismutase (SOD), peroxidase (POD), catalase (CAT), and ascorbate peroxidase (APX), along with non-enzymatic antioxidants, is responsible for scavenging reactive oxygen species (ROS) to minimize oxidative damage during periods of stress [1, 25, 29]. Numerous studies have also documented the activity of antioxidant enzymes in response to various environmental conditions [16, 32]. Malondialdehyde (MDA), a byproduct of the lipoperoxidation of cell membranes, is a biomarker of oxidative stress. It can be used to assess an individual’s susceptibility to waterlogging [18, 29].

*Sedum* species are low-maintenance succulent plants that exhibit a wide range of adaptation, effective aesthetic qualities, and drought resistance (some species can survive for up to 4 months without water) [33–35]. Furthermore, *Sedum* (Crassulaceae) is considered an ideal genus for harsh conditions in green roofs, including sun exposure, intense heat, cold, and drought [34, 35]. Although many urban landscapes often experience a lack of moisture, unpredictable rainfall can also lead to an excess of moisture, which is detrimental to the overall health of plant landscapes. The empirical performance of sedums under waterlogging varied from species to species during landscape applications, particularly under waterlogging conditions. However, it is worth noting that some species with drought resistance also have better waterlogging tolerance.

Several studies have been conducted on the drought stress of stonecrop [36–38]. However, less attention has been paid to its adaptability and adaptive mechanisms under waterlogging conditions. Previously, we revealed that *Sedum spectabile Boreau* ‘Carl’ displayed greater leaf chlorophyll content and water soluble carbohydrate concentration than the *S. spectabile* ‘Rosenteller’ after waterlogging [39]. The purpose of this research was to analyze the tolerance and characterize physiological and morphological responses in seventeen *Crassulaceae* species to waterlogging conditions. This information is essential for selecting and implementing *Crassulaceae* species in the landscape.

## Materials and methods

### Plant material and stress treatment

The vigorous cuttings of three genera of plants in *Crassulaceae* (*Hylotelephium*: S1-S8; *Phedimus*: S9-S11; *Sedum*: S12-S17) (Table 1) were planted in an aperture disk that was filled with a homogenized mixture of peat and vermiculite (v:v = 1:1) at the Beijing Forestry University nursery (40°0′ N, 116°19′ E). Two weeks later, the cuttings were transferred to plastic pots measuring 15 × 15 cm. A 2:2:1 mixture of peat, vermiculite, and sandy soil (v/v/v) was used as a substrate. Pots were randomly placed outside and irrigated every two weeks. A routine fertilization and weeding program was undertaken every two weeks to assist with the development of the treatments. All materials were purchased from Beijing Huamu Co., Ltd.

Prior to implementing waterlogging treatments, plantlets with uniform appearance and size were carefully chosen to ensure 25 replications per treatment. Using a completely randomized design, two treatments were carried out over 36 days: (1) control, in which pots were irrigated every three days, with excess water drained after each irrigation; and (2) waterlogging, in which the water level remained 2–3 cm above the soil during the treatment. Every four days, the water is properly replenished. Plantlets were allowed to grow under well-drained conditions for 12 days after being submerged to assess their recovery.

**Table 1:** Experimental plant materials

Code	Scientific name	Code	Scientific name
S1	*Hylotelephium spectabile*	S9	*Phedimus aizoon*
S2	*H. spectabile* ‘Brilliant’	S10	*P. hybridum* ‘Immergrunchen’
S3	*H. spectabile* ‘Carl’	S11	*P. selskianum* ‘Spirit’
S4	*H. spectabile* ‘Rosenteller’	S12	*S. mexicanum* ‘Gold Mound’
S5	*H. telephium* ‘Autumn Joy’	S13	*S. reflexum* ‘Blue Spruce’
S6	*H. telephium* ‘Joice’ Henderson’	S14	*S. sarmentosum*
S7	*H. telephium* ‘Purple Emperor’	S15	*S. sexangulare*
S8	*H. telephium* ‘Vera Jameson’	S16	*S. spurium* ‘Coccineum’
		S17	*S. spurium* ‘Fuldaglut’

### Survival and estimation of growth parameters

At the end of the experiment, the survival rate was recorded. Each material and treatment required the use of six plants. The production of adventitious roots was monitored every three days. At the completion of the waterlogging treatment, we counted the number and maximum length of adventitious roots, and calculated the frequency of plants with adventitious roots. Aboveground and root biomass were collected 36 days after the treatment. The dry biomass (DM) of all sections was determined by weighing after 72 h of drying at 65 °C until a constant weight was achieved. The root/shoot ratio (RS) was then calculated.

Furthermore, the height of *Hylotelephium*, which has an upright growth habit, and the coverage of *Phedimus* and *Sedum*, which have a decumbent growth habit, were measured at the beginning and end of each treatment. This data was used to calculate the relative growth rate, taking into account any variations. The leaves were then collected to determine their fresh weight (FW). Leaf discs were soaked in water for 24 h in the dark at 25 °C to obtain turgid mass (TW). There were then dried for 72 h at 65 °C to obtain the dry weight (DW) [40]. The RWC was then computed using the formula: RWC = (FW ‒ DW) × 100 / (TW ‒ DW).

### Estimation of chlorophyll concentration

During the experiments, the forth fully expanded and exposed leaves from an apex were chosen at random from treatment replications. Chlorophyll extraction (200 mg leaf FW) was determined [41], with minor modifications. After overnight extraction, the tubes were wrapped with plastic wrap and placed in a dark location until the leaf pieces turned completely white. The absorbance of the extracted liquid was then measured at 470, 645, and 663 nm using the miscible liquids as references.

### Estimation of lipid peroxidation

Fully utilize the remaining randomly collected leaves mentioned above to measure physiological parameters. The level of malondialdehyde (MDA) was measured using the thiobarbituric acid (TBA) approach [42]. 200 mg of freeze-dried material was homogenized in 5 mL of 50 mM sodium phosphate buffer (pH 7.8). The sample was then centrifuged for 20 min at 10,000 rpm. 1mL of supernatant was mixed with 1 mL of 20% (w/v) trichloroacetic acid (TCA) containing 0.5% (w/v) thiobarbituric acid (TBA). 1 mL of deionized water was mixed with 1 mL of 20% (w/v) TCA solution containing 0.5% (w/v) to create a sample blank. Both mixtures were allowed to react in a boiling water bath for 30 min before being rapidly cooled and centrifuged at 4,000 rpm for 10 min. The absorbance of the supernatant was measured at 440, 532, and 600 nm.

### Estimation of enzyme activity

The samples were homogenized in a cold sodium phosphate buffer (50 mM, pH 7.8) containing 0.1 mM EDTA, 1% (w/v) polyvinylpyrrolidone (PVP), and 1 mM dithiothreitol (DTT). The homogenate was then centrifuged at 4 °C for 10 min at 10,000 rpm. The supernatants were collected and stored at 4 °C until protein extraction and enzyme analysis. XK Wang’s approach was used to measure the activity of SOD, POD, and CAT [43].

SOD activity was measured using the photochemical NBT approach in a 3 ml test mixture. The mixture contained 50 mM phosphate buffer (pH 7.8), 13 mM l-methionine, 75 µM NBT, 10 µM EDTA-Na_2_, 2 mM riboflavin, and 0.1 mL substrate. The reaction was stopped after 20 min by removing it from the fluorescent bulb source to measure the absorbance at 560 nm. One unit of SOD was defined as the amount of enzyme that prevented 50% of NBT photoreduction. POD activity was evaluated in a 3 mL mixture containing 50 mM sodium phosphate (pH 7.0), 0.1 mM EDTA, 10 mM guaiacol, 5 mM H_2_O_2_, and 50 uL enzyme solution. The absorbance of brown guaiacol at 470 nm varied after 4 min.

Following the absorption of H_2_O_2_ at 240 nm for 40 s in a 3 ml assay mixture containing 50 mM sodium phosphate buffer (pH 7.0), 10 mM H_2_O_2_, and 0.1 ml enzyme solution, researchers were able to evaluate the catalase (CAT) activity.

### Data analysis

The data was displayed as means and standard errors. The statistical analysis was conducted using IBM SPSS 23.0, including ANOVA and the Duncan multiple range test (p ≤ 0.05). P-values less than 0.05 are considered significant for each data set, which were calculated as the mean of three replicates (n = 3).

## Results

### Waterlogging affects phenotype and survival in sedums

**Fig. 1:**
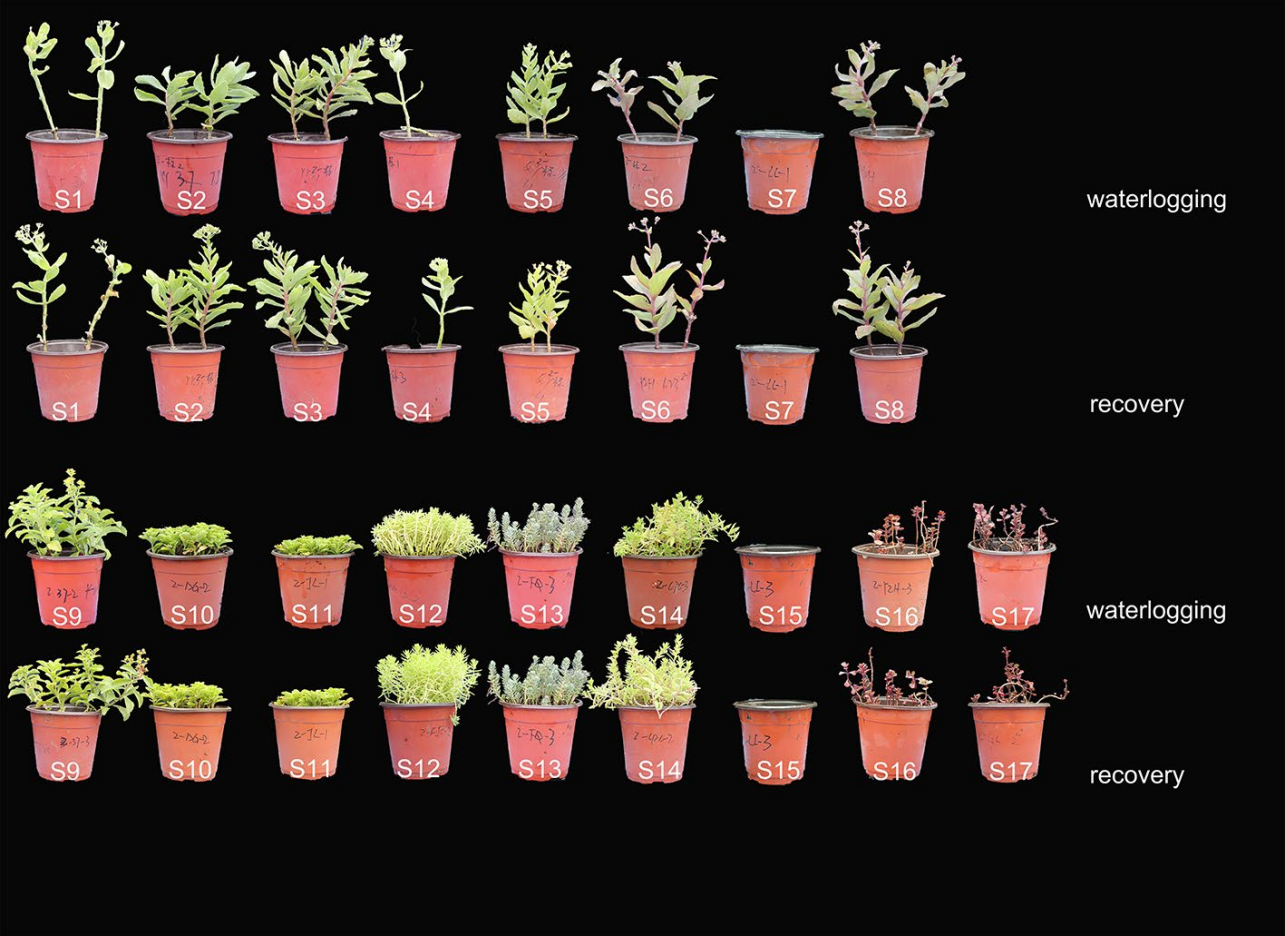
Representative pictures of 17 materials after 36 days waterlogging and 12 days recovery

After 36 days of waterlogging treatment, various responses were observed among the 17 test accessions (Fig. 1), and the survival rate of plantlets varied from 0 to 100% (Table 2). S7 and S15 exhibited clear symptoms of leaf necrosis, abscission, and stem rot within the first week. Unfortunately, neither cultivar survived beyond two weeks of waterlogging, indicating their high susceptibility to root hypoxia. As a result, the recovery treatment was limited to only 15 materials.

**Table 2:** Survival percentages of 17 materials after 36 days of waterlogging treatment

Code	Survival (%)	Code	Survival (%)	Code	Survival (%)	Code	Survival (%)
S1	100	S6	100	S11	100	S16	25
S2	100	S7	0	S12	70	S17	30
S3	100	S8	100	S13	76		
S4	50	S9	100	S14	76		
S5	100	S10	100	S15	0		

S16 and S17 exhibited significant damage to their roots due to hypoxia, resulting in death rates of 80% and 75% respectively after waterlogging. This suggests that they are highly susceptible to waterlogging. However, S2, S3, and S5 performed the best under waterlogging stress, experiencing only minor damage and successfully maintaining all of their plants. This demonstrates their exceptional tolerance to this stressful condition. S4, S13, S12, and S14 showed intermediate sensitivity to root hypoxia, as damage symptoms appeared later compared to S16, and mortality rates were 50%, 30%, 24%, and 24%, respectively. Although some foliar symptoms were observed, the remaining plants exhibited moderate tolerance to waterlogging, with no mortality observed.

After 12 days of draining, the damage symptoms improved to varying degrees (Fig. 1). New buds appeared, leaf shape and color returned to normal, and even heights increased. Flower buds also appeared in all *Hylotelephium* plants, except for S7 (data not shown).

### Adventitious root responses

Except for S7 and S15, which died early, vigorous adventitious roots had grown from the leaf scars in all species of materials, with a frequency ranging from 3 to 100% (Table 3). After 3–5 days of waterlogging, most *Hylotelephium* and *Sedum* specimens exhibited the presence of adventitious roots, while only a few adventitious roots were observed in all *Phedimus* specimens after 20 days. *Hylotelephium* also has more adventitious roots (more than 1) and a higher frequency (over 50%) compared to *Sedum* (which has approximately 3-20%, with a maximum mean of less than one of these structures per plant). Furthermore, the performance was subpar despite measuring the maximum length of the adventitious roots (3 cm) in S13.

**Table 3:** Effect of waterlogging on presence time, frequency, number and length of adventitious roots of 17 materials after 36 days of waterlogging treatment

Code	Frequency (%)	Time (day)	Number	Length (mm)	Code	Frequency (%)	Time (day)	Number	Length (mm)
S1	83	10	6.0	4.3	S9	10	20	0.1	4.6
S2	100	5	8.0	7.3	S10	10	25	0.1	2.9
S3	100	3	3.3	6.7	S11	10	22	0.1	3.5
S4	33	10	1.3	1.7	S12	20	4	0.6	4.7
S5	100	5	5.7	6.7	S13	16	5	0.8	30.0
S6	50	7.00	3.3	2.7	S14	13	3	0.96	9.3
S7	0	*np*	*np*	*np*	S15	0	*np*	*np*	*np*
S8	50	7.00	4.7	3.0	S16	3	4	0.03	2.0
					S17	7	5	0.09	7.3

*np* means no values

### MDA contents

**Fig. 2:**
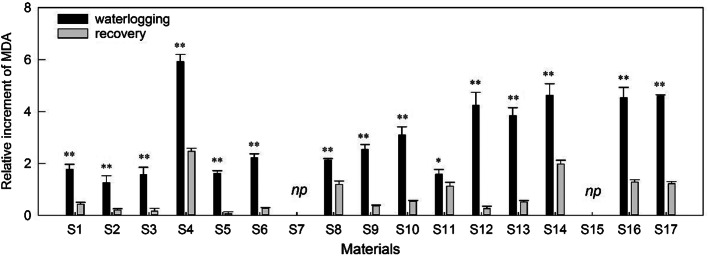
Evolution of the relative increment rate of MDA concentration in sedums compared to control during waterlogging and recovery. Vertical bars indicate standard error of the mean (n = 3), asterisk indicate significant differences at p < 0.05 according to t-test. *Np* indicates that early death and no recovery treatment

After the waterlogging treatment, all test accessions showed significant increases in MDA concentration ((p < 0.05) (Fig. 2). S2, S3, and S5 exhibited lower levels of oxidative stress injury (1.25, 1.56, and 1.6-fold, respectively), while S4 had the highest MDA contents (6.85-fold), followed by S17, S13, and S16 in descending order with comparison of well-drained conditions during waterlogging treatment.

With a few exceptions, the oxidative stress injury of materials decreased by 0.16 to 2.47-fold over the recovery period compared to their control plants (Fig. 2). During the recovery period, S2, S3, and S5 showed the highest tolerance, while S16 and S17 were the most susceptible. However, S8, S4, and S14 exhibited relatively higher content (55.81%, 42.93%, and 41.69% of the waterlogging value, respectively), indicating that subsequent drainage after waterlogging still had a negative influence on membrane stability. Th treatment, materials, and interaction between treatment and materials had a significant influence on MDA contents during both the waterlogging and recovery periods (Table 4).

**Table 4:** Results of the analysis of variance (ANOVA) of treatment (T), material (M) and their interactions for dry biomass (DM), root/Shoot ratio (RSR), relative water content (RWC), MDA, chlorophyll contents (Chl), SOD and CAT activities during waterlogging and recovery periods

Dependent variable	Independent variable					
	Waterlogging period			Recovery period		
	T	M	T×M	T	M	T×M
Dry biomass	785.8*^a^	44.9*	24.74*	2518.4*	92.3*	68.9*
Root/Shoot ratio	514.7*	50.1*	35.2*	155.0*	89.1*	30.0*
Relative water content	6696.4*	99.0*	96.3*	122.8*	14.3*	7.5*
MDA	1069.5*	34.7*	21.2*	359.9*	64.2*	17.5*
Chl(a + b)	463.1*	37.2*	4.4*	66.8*	43.3*	4.7*
SOD	2001.0*	35.6*	14.6*	1816.3*	12.6*	7.9*
CAT	3134.3*	92.6*	74.8*	1581.2*	51.5*	38.3*

^*^Significant at p ≤ 0.05

^a^Numbers represent F-values at 5% level

### Growth parameters

**Fig. 3:**
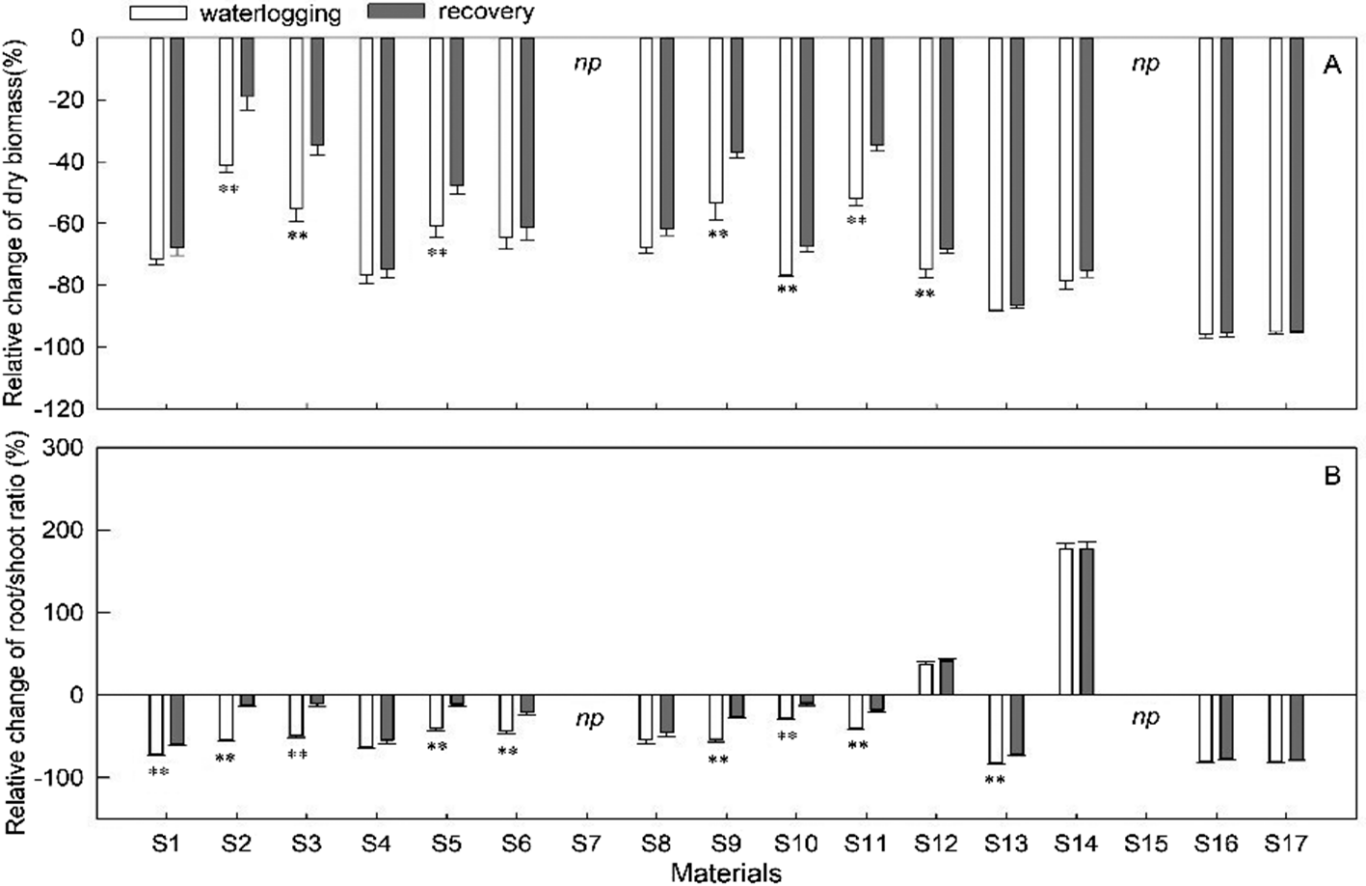
Evolution of the increment rate of dry biomass and root-shoot ratio in sedums compared to control during 36 days of waterlogging followed by 12 days of recovery. Vertical bars indicate standard error of the mean (n = 3), asterisk indicate significant differences at p < 0.05 according to t-test. *Np* indicates that early death and no recovery treatment

Waterlogging significantly reduced total dry biomass and root/shoot ratios significantly (p<0.05) (Fig. 3). Both parameters declined more dramatically in S16 and S17, with reductions of approximately 95% and 80%, respectively (Fig. 3). However, in various species, the decreases were less significant. When compared to the control, S2, S3, S9, and S11 exhibited lower relative total dry biomass decrement values (41%, 55%, 53%, and 52%, respectively) (Fig. 3A). Similarly, the root-shoot ratios decreased by 28% in S10 and 37% in S12. Furthermore, there was a significant improvement of 177% in S14 and 37% in S12 waterlogged plants was seen as compared to the control. The remaining materials exhibited moderate decreases in root-shoot ratios. In addition, the severity of root injury was greater than that of shoot injury, indicating that root injury is a major cause of waterlogging stress.

Following the periods of waterlogging, species showed different patterns of biomass accumulation and allocation (Fig. 3). Plantlets were able to resume energetic growth after waterlogging, especially in their roots (Fig. 3B). This allowed S2, S3, S5, S9, and S11 to recover more quickly than waterlogged plants, not only in terms of dry biomass (with only a 19–35% decline rate) (Fig. 3A), but also in their root/shoot ratio (with only a 9–12% decline rate) (Fig. 3B). However, S16 and S17 exhibited the most significant decreases in dry biomass and root/shoot ratio, indicating that the adverse effects of anoxia injury on dry biomass accumulation and allocation persisted until the end of the experiment, with only a slight increase in comparison to their respective controls. Meanwhile, the other materials were in the intermediate stage. After 12 days of recuperation, the root/shoot ratio increased, indicating that carbohydrates were preferentially transferred to the root system. Furthermore, there was a significant impact of treatment, materials, and the combination of treatment and materials on dry biomass and root/shoot ratio during both the waterlogging and recovery periods (Table 3).

### RWC contents

**Fig. 4:**
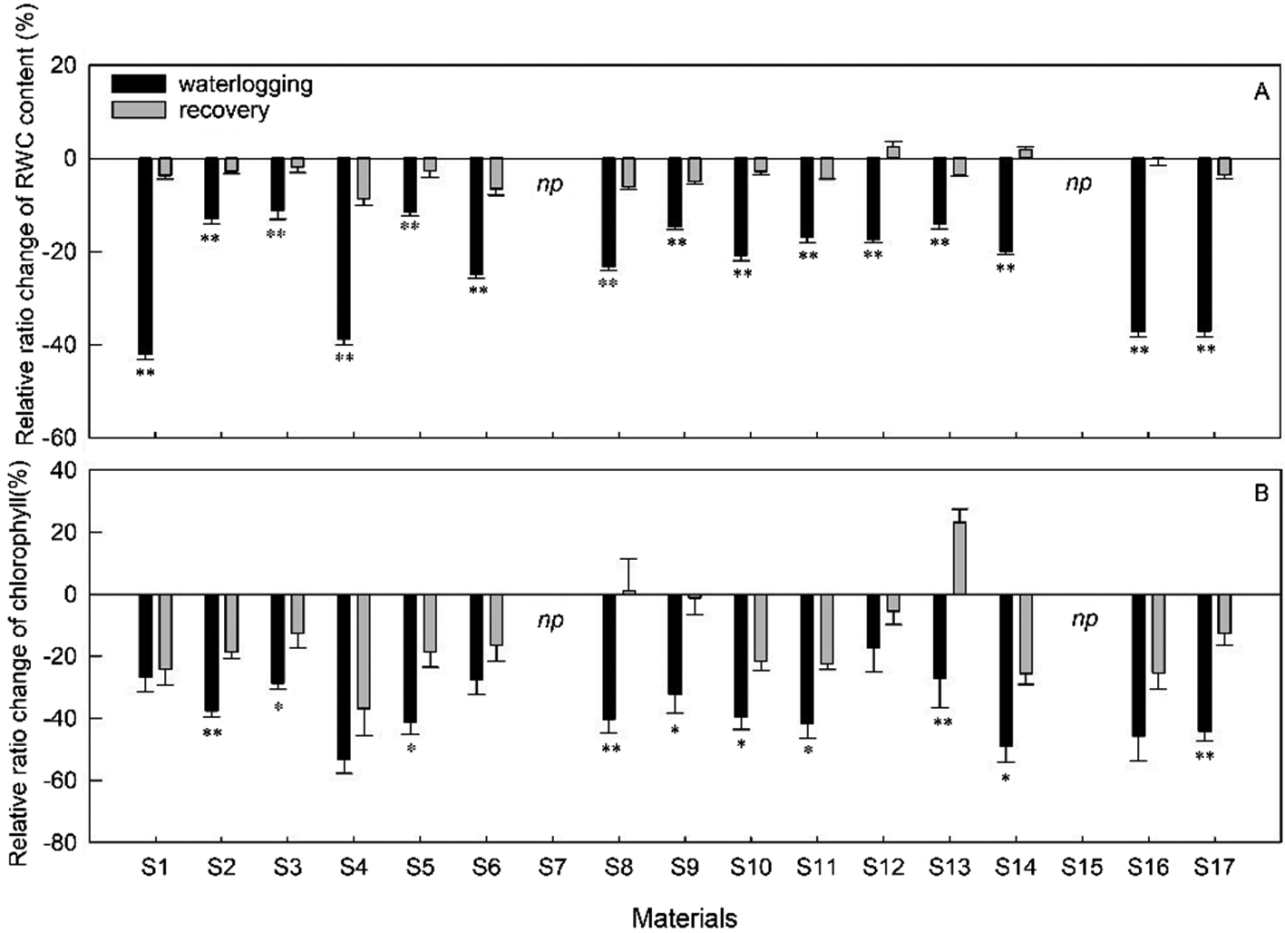
Evolution of the increment rate of RWC content and Chl content in sedums compared to control during 36 days of waterlogging followed by 12 days of recovery. Vertical bars indicate standard error of the mean (n = 3), asterisk indicate significant differences at p < 0.05 according to t-test. *Np* indicates that early death and no recovery treatment

Prolonged periods of waterlogging decreased the relative water content (RWC), although the extent of reduction varied depending on the materials (Fig. 4A). S2, S3, S5, and S9 exhibited only a 15% reduction in relative water content (RWC) compared to their respective controls, while S1, S4, S16, and S17 showed a 37%–42% decrease in RWC content compared to their controls.

Following the period of waterlogging, the materials showed different levels of recovery in terms of RWC content (Fig. 4A). Except for a 9% reduction in RWC in S4, the majority of materials reached to a level similar to that of the non-stressed controls. Furthermore, the levels of S12 and S14 increased slightly compared to the controls.

### Photosynthetic pigments

The waterlogging treatment significantly reduced the total chlorophyll content (p ≤ 0.05) compared to the control (Fig. 4B). Total chlorophyll concentration dropped considerably across all cuttings grown in waterlogged conditions, with levels ranging from 16 to 53% lower than the corresponding control (Fig. 4B). During the experiment, the lowered chlorophyll concentration in S12 was substantially lower than in the other samples, showing only a 17% loss compared to the control. Furthermore, the leaf Chl concentration reduced by 53% and 50% in waterlogged S4 and S14 plants, respectively. After 12 days of draining, the chlorophyll content in stressed plants increased in all materials, reaching levels higher than in control plants for S8 and S13, but not for S1, S4, S6, S12, and S16.

### Enzyme activity

**Fig. 5:**
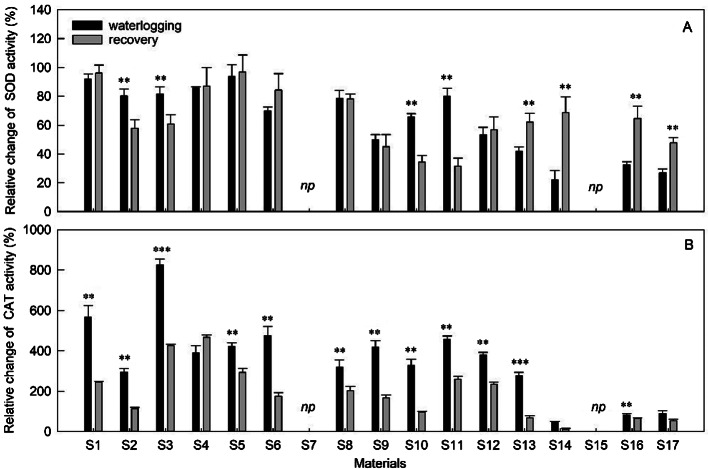
Evolution of the increment rate of SOD and CAT activity in sedums compared to control during 36 days of waterlogging followed by 12days of recovery. Vertical bars indicate standard error of the mean (n = 3), asterisk indicate significant differences at p < 0.05 according to t-test. *Np* indicates that early death and no recovery treatment

The application of waterlogging resulted in varied responses in different cuttings in terms of antioxidative enzyme activities, such as SOD and CAT, which are crucial in combating the harmful effects of ROS (Fig. 5). SOD showed higher basal levels of the two antioxidant enzymes compared to CAT. In comparison to the control plants, SOD activity considerably climbed under waterlogging conditions (p < 0.05) (Fig. 5A). S14, S16, and S17 have lower SOD levels than other species, with an increase of only 22–33%. The sensitive species S4 (*H. spectabile* ‘Rosenteller’) responded to flooding by increasing SOD activity, and after 36 days of treatment, the level was 86% higher than that of control plants. Additionally, during the recovery period, the variation in SOD activity differs among sedums (Fig. 5A). The SOD activity of S2, S3, S10, and S11 significantly decreased, while it significantly increased in S12, S14, S16, and S17. This indicates that subsequent drainage after waterlogging worsened rather than improved the performance of sensitive materials.

In comparison to control plants, waterlogging generated a considerable increase in CAT activity, showing a significant and positive correlation with SOD (p < 0.001, r = 0.70) (Fig. 5A). Particularly, the values of CAT activities demonstrated higher values compared to SOD, ranging from at least 1.5 to 8.8-fold higher than those discovered in their respective control plants (Fig. 5B). Even though S1 (*H. spectabile*) had unacceptably poor phenotypic quality after 36 days of waterlogging treatment, the CAT activity was higher than most of species with 6.5 times compared with control plants, just lower than the highest CAT activity of S3 (*H. spectabile* ‘Carl’, 8.8-fold). All sedums, except for S4, S14, and S17, showed a decrease in CAT activity following the recovery period, while S4 exhibited a minor increase. Furthermore, in both treatment periods, a two-way ANOVA demonstrated a significant impact of the therapy, material, and the interaction between the treatment and material.

### Diversity of morpho-physiological responses to waterlogging as a grouping criterion

To investigate the morpho-physiological response associated with tolerance, a similarity-grouping analysis was performed using the ratio between waterlogged and control values of the following attributes (Table 5). All of the mean values of S2, S3, S5, and S9 were greater than 0.72, indicating that waterlogging resulted in less damage. However, the average values of S4, S7, S15, S16, and S17 were lower than 0.50, indicating that their development was significantly hindered by waterlogging. According to the findings, S2, S3, S5, and S9 exhibited tolerance to waterlogging, while S4, S16, and S17 were susceptible to waterlogging. S7 and S15 were the most sensitive materials, with the tolerance of the remaining materials falling between them.

**Table 5:** Subordinate function values of different sedums under waterlogging stress and evaluation of waterlogging tolerance

Code	Survival	Dry biomass	Root/shoot ratio	RWC	MDA	Chl t	SOD	POD	average	Score	
S1	1.00	0.48	0.10	0.65	0.60	0.84	1.00	0.68	0.67	8	
S2	1.00	1.00	0.16	0.98	0.67	0.84	0.93	0.42	0.75	2	
S3	1.00	0.77	0.18	1.00	0.64	1.00	0.94	1.00	0.82	1	
S4	0.50	0.40	0.14	0.69	0.00	0.62	0.96	0.53	0.48	13	
S5	1.00	0.67	0.21	1.00	0.62	0.79	1.00	0.56	0.73	3	
S6	1.00	0.59	0.20	0.84	0.53	0.97	0.88	0.62	0.70	5	
S7	0.00	0.00	0.00	0.00	0.00	0.00	0.00	0.00	0.00	16	
S8	1.00	0.55	0.16	0.86	0.54	0.77	0.92	0.45	0.66	9	
S9	1.00	0.80	0.16	0.96	0.59	0.91	0.78	0.55	0.72	4	
S10	1.00	0.40	0.25	0.89	0.40	0.81	0.86	0.46	0.63	10	
S11	1.00	0.82	0.21	0.93	0.24	0.78	0.93	0.60	0.69	6	
S12	0.70	0.43	0.48	0.93	0.62	1.00	0.79	0.52	0.68	7	
S13	0.76	0.20	0.06	0.97	0.14	0.97	0.74	0.40	0.53	12	
S14	0.76	0.36	1.00	0.90	0.23	0.69	0.63	0.16	0.59	11	
S15	0.00	0.00	0.00	0.00	0.00	0.00	0.00	0.00	0.00	17	
S16	0.25	0.07	0.07	0.71	0.20	0.72	0.69	0.20	0.36	15	
S17	0.30	0.08	0.07	0.71	0.18	0.83	0.66	0.21	0.38	14	

**Fig. 6:**
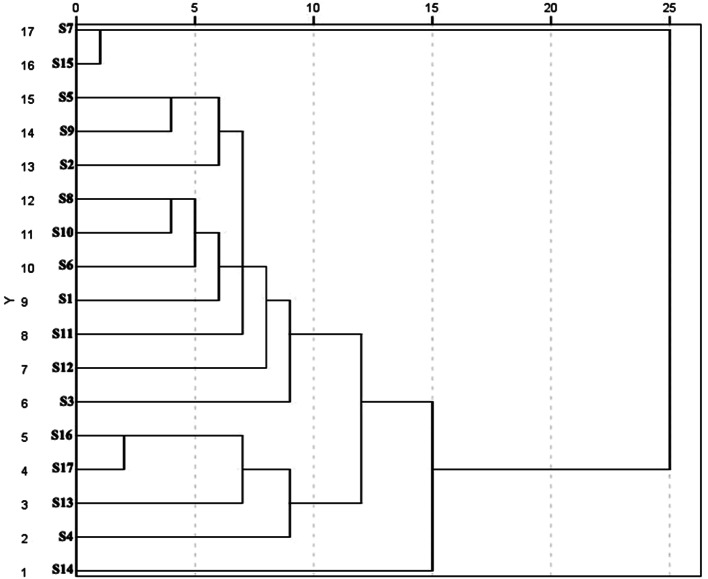
UPGMA dendrograms of sedums constructed based on waterlogging tolerance coefficients. The similarity cutoff for definition of groups is indicated by a dashed vertical line

Based on the pooled data, the cluster analysis revealed that 17 species were divided into four clusters, with 10, 4, 2, and 1 species in each cluster (Fig. 6). The first group included S1, S2, S3, S5, S6, S8, S9, S10, S11, and S12, all of which were waterlogged tolerant. S14, which is somewhat resistant to waterlogging, belongs to the second group. S4, S13, S16, and S17 were in the third group, which experienced mild waterlogging sensitivity. S7 and S15 were the most susceptible to waterlogging.

## Discussion

Throughout the waterlogging experiment, we assessed the tolerance of seventeen sedum species to prolonged root hypoxia. All test accessions differed not only in their response to waterlogging but also in the time it took for them to recover. The formation of adventitious roots has been mentioned as a potential strategy to avoid hypoxia resulting from waterlogging [16]. this process may facilitate in the absorption of water, oxygen, and nutrients, alleviate hypoxic conditions [21], and enhance the plant’s ablity to tolerate and survive or recover from waterlogging conditions [28]. The findings revealed the presence of adventitious roots at the leaf scar of *Hylotelephium* and *Sedum* specimens after 3 days of waterlogging (Table 2). Although the range of formation time and root numbers varies between species, these roots appear to have been crucial for the survival of sedums, as indicated by their ability to 36 days of waterlogging. The formation of abundant adventitious roots in S1’s leaf scar, in particular, may contribute to its unsatisfactory phenotypic quality without causing death. Furthermore, despite the absence of adventitious roots in three *Phedimus* materials, their high tolerance suggests the existence of additional morpho-anatomical adaptation mechanisms, such as aerenchyma development and radial oxygen-loss barrier construction [44].

Waterlogging has been described as causing predominant inhibition of biomass accumulation and survival [8, 28, 39]. The dry biomass and root-shoot ratio of 17 materials were obviously lowered by prolonged waterlogging compared to the control (Fig. 1), indicating that waterlogging conditions hindered the development of sedums, although most plants survived. Furthermore, the level of damage produced by waterlogging stress varied significantly across the 17 materials. Many water-tolerant species have a decreased root-shoot ratio when waterlogged [16, 44]. However, the root-shoot ratio of S12 and S14 increased (Fig. 3B), which contradicts the previously mentioned trend. This could be due to a greater loss of aboveground dry matter compared to root dry matter.

Plants have the ability to slow down their metabolism to conserve energy and carbohydrates for later use, allowing for a resumption of vigorous growth during recuperation [9]. The recovery phase, which is a crucial period for assessing the true waterlogging tolerance of specific species, revealed varying degrees of ability to recover for different species [45]. The degree of preferential biomass allocation toward roots during recovery varied among sedums in the current investigation (Fig. 3A). Particularly in S2, S3, S5, S9, and S11, the biomass toward roots enables the re-establishment of a more suitable shoot-to-root ratio for facilitating water and nutrient supply in well-drained soil [45]. However, the poor biomass recovery of S16 and S17 after 12 days of water subsided indicates that they were considerably stressed by waterlogging, and that the damage to their root systems could not be repaired in the near term (12 days). S2, S3, S5, S9, and S11 consistently showed greater relative growth than other materials during periods of waterlogging and recovery.

Adequate water content is beneficial for stabilizing subcellular structures and aiding cell recovery from stress. Waterlogging often reduces plant RWC [29, 32], as demonstrated in the current study. Prolonged waterlogging significantly reduced the relative water content (Fig. 4A), with the most obvious drop observed in S16 and S17, and the least pronounced in S2, S3, S5, and S9. Interestingly, recovery over 12 days led to a significant decrease in RWC in all plants, even though the levels of most plants were close to their controls. This indicates that rapid restoration of RWC upon removal of waterlogging stress may contribute to resistance acquisition.

In the present study, waterlogging significantly decreased the leaf chlorophyll content, and after a period of recovery, the chlorophyll content climbed for all materials, although to varying degrees (Fig. 4B). This is consistent with findings in *Rhododendron delavay* [11] and *Actinidia valvata* Dunn [25]. The decrease in chlorophyll concentration produced by environmental stress is related to the suppression of synthesis and faster degradation of chlorophyll in order to prevent photo-oxidation [14]. Durhman et al. (2006) and Kozminska at al. (2019) discovered that Chl responses to drought stress contribute to tolerance, which is consistent with the current study showing tolerant sedums have superior adaptive instincts than sensitive ones [33, 46]. During waterlogging, sedums with lower Chl content loss were observed to accumulate organic reserves, which increased their resilience after the stress subsided. This effect was confirmed by the increased dry biomass of tolerant materials after the recovery period (Fig. 3A).

The MDA content, which is considered a measure of oxidative damage in plant tissues under abiotic stress, was significantly increased in the present study, indicating damage to cell membranes caused by waterlogging (Fig. 3a). However, the lower concentration of MDA in tolerant species suggests that they have a greater capacity to eliminate ROS and maintain high membrane stability compared to sensitive species [39]. Waterlogging in sedums can cause damage to the cell membrane, leading to a reduction in photosynthesis and chlorophyll degradation [28]. However, inducing antioxidant enzyme activities to scavenge ROS can help alleviate oxidative damage associated with waterlogging and is positively related to the degree of tolerance [16, 32]. This suggests that tolerant species may activate a series of antioxidant enzymes to alleviate oxidative damage and exhibit lower basal MDA content than sensitive ones.

Previous studies have shown that tolerant sedums are able to maintain higher levels of antioxidant enzymes such as SOD, POD and CAT compared to sensitive sedums under water stress (Fig. 5). This allows them to maintain a better balance between ROS formation and detoxification, which is consistent with our own findings [16]. Specifically, our study observed a higher level of antioxidant enzyme activities induced by waterlogging and identified a positive correlation between these activities (Fig. 5). These results suggest that sedums are equipped with efficient antioxidative systems that can protect them from oxidative injury caused by waterlogging. It is noteworthy that CAT activities rose more than SOD (Fig. 5), suggesting that H_2_O_2_ scavenging by CAT is one of the most essential mechanisms for sedums to protect against waterlogged stress.

## Conclusions

In conclusion, all test accessions performed significant differences in waterlogging tolerance and recovery capability. Most sedums, with the exception of the most sensitive varieties such as *H. telephium* ‘Purple Emperor’ and *S. sexangulare*, can withstand up to 36 days of continuous waterlogging and have good recovery abilities. This indicates that they are desirable species for the construction of rain gardens or impeded drainage. This is the second study of sedums’ morphological and physiological responses to waterlogging. We believe that the findings of this study will not only contribute to the development of landscaping applications aimed at selecting waterlogging-adapted plants, but also enhance our understanding of the waterlogging stress response in plants.

## Data Availability

Some data used in this study were included within the article. Other data are available from corresponding authors, please contact 349214940@qq.com.

## References

[1] Tyagi A, Ali S, Park S, Bae H. Exploring the potential of multiomics and other integrative approaches for improving waterlogging tolerance in plants. Plants-Basel. 2023;12(7). 10.3390/plants12071544. 10.3390/plants12071544 PMC10096958 37050170

[2] Habibi F, Liu T, Shahid MA, Schaffer B, Sarkhosh A. Physiological, biochemical, and molecular responses of fruit trees to root zone hypoxia. Environ Exp Bot. 2023;206. 10.1016/j.envexpbot.2022.105179.

[3] Pan J, Sharif R, Xu X, Chen X (2021). Mechanisms of waterlogging tolerance in plants: research progress and prospects. Front Plant Sci.

[4] Sprunger CD, Lindsey A, Lightcap A (2023). Above- and belowground linkages during extreme moisture excess: leveraging knowledge from natural ecosystems to better understand implications for row-crop agroecosystems. J Exp Bot.

[5] Team CW, Lee H, Romero J, IPCC., 2023: Sections. In: Climate Change 2023: Synthesis Report. Contribution of Working Groups I, II and III to the Sixth. Assessment Report of the Intergovernmental Panel on Climate Change, Geneva, Switzerland: IPCC; 2023.

[6] Pedersen O, Sauter M, Colmer TD, Nakazono M (2020). Regulation of root adaptive anatomical and morphological traits during low soil oxygen. New Phytol.

[7] Daniel K, Hartman S (2023). How plant roots respond to waterlogging. J Exp Bot.

[8] Qiu CS, Qiu HJ, Peng DX, et al. The mechanisms underlying physiological and molecular responses to waterlogging in flax. J Nat Fibers. 2023;20(2). 10.1080/15440478.2023.2198275.

[9] Voesenek LACJ, Bailey-Serres J (2008). Flooding stress: acclimations and genetic diversity. Annu Rev Plant Biol.

[10] Xie LJ, Zhou Y, Chen QF, Xiao S. New insights into the role of lipids in plant hypoxia responses. Prog Lipid Res. 2021;81. 10.1016/j.plipres.2020.101072. 10.1016/j.plipres.2020.101072 33188800

[11] Zhang XM, Duan SG, Xia Y, Li JT, Liu LX, Tang M, Tang J, Sun W, Yi Y. Transcriptomic, physiological, and metabolomic response of an alpine plant, *Rhododendron delavayi*, to waterlogging stress and post-waterlogging recovery. Int J Mol Sci. 2023;24(13). 10.3390/ijms241310509. 10.3390/ijms241310509 PMC10341954 37445685

[12] Hasanuzzaman M, Bhuyan MHMB, Zulfiqar F, Raza A, Mohsin SM, Mahmud JA, Fujita M, Fotopoulos V (2020). Reactive oxygen species and antioxidant defense in plants under abiotic stress: revisiting the crucial role of a universal defense regulator. Antioxidants.

[13] Luo J, Yu W, Xiao Y, Zhang Y, Peng F (2022). Strawberry *FaSnRK1α* regulates anaerobic respiratory metabolism under waterlogging. Int J Mol Sci.

[14] Wu J, Wang J, Hui W, Zhao F, Wang P, Su C, Gong W (2022). Physiology of plant responses to water stress and related genes: a review. Forests.

[15] Shi FH, Pan ZJ, Dai PF, Shen YB, Lu YZ, Han B. Effect of waterlogging stress on Leaf Anatomical structure and ultrastructure of *Phoebe Sheareri* Seedlings. Forests. 2023;14(7). 10.3390/f14071294.

[16] Zhao T, Pan X, Ou Z, Li Q, Zhang WE (2022). Comprehensive evaluation of waterlogging tolerance of eleven *Canna* cultivars at flowering stage. Sci Hortic.

[17] Zhang H, Li G, Yan C, Zhang X, Cao N, Le M, Hu X, Zhu F, Liu W (2022). Elucidating the molecular responses to waterlogging stress in *Cucumis melo* by comparative transcriptome profiling. Horticulturae.

[18] He W, Luo L, Xie R, et al. Transcriptome sequencing analyses uncover mechanisms of citrus rootstock seedlings under waterlogging stress. Front Plant Sci. 2023;14. 10.3389/fpls.2023.1198930. 10.3389/fpls.2023.1198930 PMC10264899 37324702

[19] Ploschuk RA, Miralles DJ, Striker GG (2023). Waterlogging tolerance of winter crops: Root mass density and canopy dynamics. Agron J.

[20] Pimentel P, Almada R, Salvatierra A, Toro G, Arismendi MJ, Pino M, Sagredo B, Pinto M (2014). Physiological and morphological responses of *Prunus* species with different degree of tolerance to long-term root hypoxia. Sci Hortic.

[21] Parent C, Nicolas C, Audrey B, Crevècoeur M, Dat J (2008). An overview of plant responses to soil waterlogging. Plant Stress.

[22] Guidi L, Lo Piccolo E, Landi M. Chlorophyll fluorescence, photoinhibition and abiotic stress: does it make any difference the fact to be a C3 or C4 species? Front Plant Sci. 2019;10. 10.3389/fpls.2019.00174. 10.3389/fpls.2019.00174 PMC6382737 30838014

[23] Armstrong W, Beckett PM, Colmer TD, Setter TL, Greenway H (2019). Tolerance of roots to low oxygen: ‘Anoxic’ cores, the phytoglobin-nitric oxide cycle, and energy or oxygen sensing. J Plant Physiol.

[24] Kitomi Y, Hanzawa E, Kuya N, Inoue H, Uga Y (2020). Root angle modifications by the *DRO*_1_ homolog improve rice yields in saline paddy fields. Proc Natl Acad Sci.

[25] Gao MX, Gai CY, Li XY, Feng X, Lai RL, Song YY, Zeng RS, Chen DQ, Chen YT. Waterlogging tolerance of *Actinidia Valvata* Dunn is associated with high activities of pyruvate decarboxylase, alcohol dehydrogenase and antioxidant enzymes. Plants-Basel. 2023;12(15). 10.3390/plants12152872. 10.3390/plants12152872 PMC10421509 37571025

[26] Arif M, Rauf M, Awais M, Ud-Din A, Hamayun M (2021). Molecular mechanisms of the 1-aminocyclopropane-1-carboxylic acid (ACC) deaminase producing *Trichoderma Asperellum* MAP1 in enhancing wheat tolerance to waterlogging stress. Front Plant Sci.

[27] Sairam RK, Dharmar K, Chinnusamy V, Meena RC (2009). Waterlogging-induced increase in sugar mobilization, fermentation, and related gene expression in the roots of mung bean (*Vigna radiata*). J Plant Physiol.

[28] Evans DE, Gladish DK. Plant responses to waterlogging. In: Encyclopedia of Applied Plant Sciences (Second Edition). Oxford: Academic Press; 2017: 36–39. 10.1016/B978-0-12-394807-6.00083-6.

[29] Balakhnina TI (2015). Plant responses to soil flooding. Stress responses in plants: mechanisms of toxicity and tolerance.

[30] Striker GG, Colmer TD (2017). Flooding tolerance of forage legumes. J Exp Bot.

[31] Finkel T, Holbrook NJ (2000). Oxidants, oxidative stress and the biology of ageing. Nature.

[32] Haddadi BS, Hassanpour H, Niknam V (2016). Effect of salinity and waterlogging on growth, anatomical and antioxidative responses in *Mentha aquatica* L. Acta Physiol Plant.

[33] Durhman AK, Rowe DB, Rugh CL (2006). Effect of watering regimen on chlorophyll fluorescence and growth of selected green roof plant taxa. HortScience.

[34] Pérez G, Chocarro C, Juárez A, Coma J. Evaluation of the development of five *Sedum* species on extensive green roofs in a continental Mediterranean climate. Urban for Urban Green. 2020;48. 10.1016/j.ufug.2019.126566.

[35] Seyedabadi MR, Karrabi M, Nabati J (2022). Investigating green roofs’ CO_2_ sequestration with cold- and drought-tolerant plants (a short- and long-term carbon footprint view). Environ Sci Pollut Res Int.

[36] Pérez G, Chocarro C, Juárez A, Coma J (2020). Evaluation of the development of five *Sedum* species on extensive green roofs in a continental Mediterranean climate. Urban for Urban Green.

[37] Nektarios PA, Kokkinou I, Ntoulas N (2020). The effects of substrate depth and irrigation regime, on seeded Sedum species grown on urban extensive green roof systems under semi-arid Μediterranean climatic conditions. J Environ Manage.

[38] Richards J, Cooke EL, Coombes M, Jones J, Viles H (2023). Evaluating the robustness of nature-based solutions: future resilience of sedum-based soft capping as a conservation approach for heritage sites in Britain and Ireland. Phys Geogr.

[39] Zhang J, Yin DJ, Fan SX, Li SG, Dong L (2019). Modulation of morphological and several physiological parameters in *sedum* under waterlogging and subsequent drainage. Russ J Plant Physiol.

[40] Weatherley PE (2010). Studies in the water relations of the cotton plant 1. The field measurement of water deficits in leaves. New Phytol.

[41] Knudson LL, Tibbitts TW, Edwards GE (1977). Measurement of ozone Injury by determination of leaf chlorophyll concentration. Plant Physiol.

[42] Arbona V, Hossain Z, López-Climent MF, Pérez-Clemente RM, Gómez-Cadenas A (2008). Antioxidant enzymatic activity is linked to waterlogging stress tolerance in citrus. Physiol Plant.

[43] Wang X (2016). The principle and technology of plant physiology and biochemistry experiment.

[44] Tuheteru FD, Kusmana C, Mansur I, Iskandar (2015). Response of lonkida (*Nauclea Orientalis* L.) towards mycorrhizal inoculum in waterlogged condition. Biotropia.

[45] Striker GG (2012). Time is on our side: the importance of considering a recovery period when assessing flooding tolerance in plants. Ecol Res.

[46] Kozminska AH, MohamadWiszniewska, AlinaHanus-Fajerska. EwaBoscaiu, MonicaVicente, Oscar. Responses of succulents to drought: comparative analysis of four sedum (*Crassulaceae*) species. Sci Hortic. 2019;243. 10.1016/j.scienta.2018.08.028.

